# Monetization in online streaming platforms: an exploration of inequalities in Twitch.tv

**DOI:** 10.1038/s41598-022-26727-5

**Published:** 2023-01-20

**Authors:** A. Houssard, F. Pilati, M. Tartari, P. L. Sacco, R. Gallotti

**Affiliations:** 1 CHuB Lab, Fondazione Bruno Kessler, Via Sommarive 18, 38123 Povo, Italy; 2 IULM University, Via Carlo Bo 1, 20143 Milan, Italy; 3 ISPC, CNR, Via Cardinale Guglielmo Sanfelice 8, 80134 Naples, Italy; 4 DiSFiPEQ, University of Chieti-Pescara, Viale Pindaro 42, 65127 Pescara, Italy; 5 Present Address: Centre Internet et Société (UPR 2000), CNRS, Paris, France

**Keywords:** Human behaviour, Scientific data

## Abstract

The live streaming platform Twitch underwent in recent years an impressive growth in terms of viewership and content diversity. The platform has been the object of several studies showcasing how streamers monetize their content via a peculiar system centered around para-sociality and community dynamics. Nonetheless, due to scarcity of data, lots is still unknown about the platform-wide relevance of this explanation as well as its effect on inequalities across streamers. In this paper, thanks to the recent availability of data showcasing the top 10,000 streamers revenue between 2019 and 2021, as well as viewership data from different sources, we characterized the popularity and audience monetization dynamics of the platform. Using methods from social physics and econometrics, we analyzed audience building and retention dynamics and linked them to observed inequalities. We found a high level of inequality across the platform, as well as an ability of top streamers to diversify their revenue sources, through audience renewal and diversification in monetization systems. Our results demonstrate that, even if the platform design and affordance favor monetization for smaller creators catering to specific niches, its non-algorithmic design still leaves room for classical choice biases allowing a few streamers to emerge, retain and renew a massive audience.

## Introduction

Twitch is a live streaming platform that allows individuals to broadcast different types of content, and viewers to react and interact with each other and with creators. While Twitch allows people to produce and upload content related to any kind of activity (online and offline), it was, and still is, primarily focused on gaming activities and culture, and can be considered the largest online gaming community in history^1^.

As Taylor^2^ puts it, Twitch users “transform their private play into public entertainment”. The motivations of viewers of streamed online gaming are more socially oriented than those of traditional media, the more so the less populated their preferred viewing channels, where a stronger social engagement can be attained with respect to more populated channels^3^. While social connection seems to be one of the primary motivations, other factors such as stress release or information seeking play a role^4^. In view of such a large and motivated user base, it is not surprising that the link between gaming culture and Twitch is deeply embedded in every aspect of the platform^5^, which is characterized by a highly specific social and revenue affordances, among others^6,7^. In just a few years, Twitch has become a cornerstone of gaming culture, allowing amateur players to turn their passion into a job^8^, boosting the development of the eSports scene^9^, and providing a venue for different gaming communities to come together^10^.

For many gamers, Twitch has become a recreational mode of consumption that is extremely common and integrated into their everyday lives^11^. Twitchers use the platform as an affectively charged backdrop for their gameplay^12^, as well as a place for small groups to gather and interact^13^, and as a delivery point of exciting, massive live action streams^14^. The collective effervescence of engrossing eSport events compares to that of traditional, large scale cultural and media ones^15^.

As mentioned, one of the most striking aspects of this connection to the gaming community is the prevalence of public social activities^16^, which echo and complement the kind of direct interactions allowed and encouraged in most online games^17^.

This social demand has shaped one of Twitch’s most relevant features: the chat. Indeed, each stream incorporates a chat that allows synchronous interaction between viewers and with the streamers themselves. It is mostly through this feature that streamers are able to engage in para-social interactions^18^, build their communities^19^, and nudge them toward the monetization features provided by the platform^20^.

Such trends have been extensively characterized by several authors^21–23^ and have been conducive to a crucial breakthrough in understanding the monetization system of the platform^24^. Nonetheless, the study of the monetization mechanisms and the logic that drives the actors in the market are mainly based on qualitative or survey materials^25^, and thus have not been able to describe the large scale dynamic related to content monetization in Twitch.

In addition, Twitch gained much popularity during the Covid-19 pandemic^26^, more than doubling the platform’s audience from 1.26 million average concurrent viewers in 2019 to 2.78 million in 2021^27^, making the study of the global market momentum of the platform even more urgent^28^, while at the same time highlighting the platform’s potential in tracking and characterizing emergent social trends among the young^29^.

Moving from this urgency, the goal of this paper is to apply computational social science tools to unveil what are the mechanisms of monetization in Twitch and explain in parallel the social dynamics embedded in the platform that enables such market system. The importance of our findings is twofold. On the one hand, studying Twitch as a social phenomenon, in view of its enormous importance in the current digital ecosystem, is relevant in itself. On the other hand, certain features of Twitch make this very platform a case study of unique interest. Twitch is in fact one of the few digital platforms that does not use any algorithmic recommendation system (other than simple random suggestion mechanics or the mere display of one’s chronology), and in parallel it is still one of the few social media that has an in-built monetization system for streamers. The combination of these two features is therefore of extreme interest not only to understand the inner logic of the platform, but also to be able to gain some deeper insight about the emerging challenges as to the use of the aforementioned technologies in digital society, in particular about economic and social value creation and extraction as related to surveillance, monitoring and algorithmic profiling^30^.

Thanks to a recent data leak suffered by Twitch, we were able to access native information from the platform itself (and thus not relying on external sources), reporting the revenue of the top 10,000 streamers. The availability of such information, that we access not directly but through publicly available lists compiled by journalists and data analysts^31^, allowed us to analyze the monetization patterns on the platform and to re-use all of the other available data in order to reach a robust and generalizable result. Such data allow us to parse the monetization system within the platform and, specifically, to evaluate inequality across streamers and the processes leading to its emergence and consolidation.

### Navigating and socializing on Twitch

We mentioned that Twitch was built with the video game subculture in mind when designing its features. Here, we will examine in greater detail some of the most relevant features and possibilities for stream monetization.

Although Twitch is not limited to gaming, the technical construction of the platform and all of its affordances relate to this subculture. For example, Hamilton, Garretson et Kerne^32^ show that one of the main motivations for consuming content produced on Twitch is a sense of belonging to the gamer community, and Cocq^22^ notes the openness and meritocratic nature of live streaming funding. Such preponderance of the gaming subculture is reflected in the way website navigation is organized.

First, content segmentation occurs according to the game currently played by the streamers, and each stream is further categorized according to gameplay type, level, etc. In addition, here we see an innovative affordance of the navigation, with an ever-present “followed channels” bar that allows users to easily navigate through known streams and the related recommendations list in the background that invites viewers to visit previous streams.

Once on a stream page, Twitch offers a unique mix of “hot and cool media”^32^, combining a high-fidelity video flux with a low-fidelity chat. This mix strongly encourages interaction among viewers, who can comment upon, and react to, the content delivered by the streamer. In addition, the streamer’s classic configuration, which uses a microphone and a webcam in addition to gameplay, allows for the deployment of further top-down connectivity.

Those ideas translate into specific features aimed at facilitating navigation. Viewers can become followers of any given channel, keeping track of active streamers at any moment and getting alerts when they go live. Highly engaged viewers can become subscribers, paying monthly amounts to support a stream, gaining privileges and rewards that make them feel part of the community. Finally, viewers can become donors who directly give money to the streamer. Specifically, viewers gain “channel points” that can be used to influence the stream in some ways, giving them agency privilege. Moreover, subscribers get badges associated to their name testifying to their membership status, as well as channel specific emotes. All such features encourage viewers to come back to the same stream, deeply engage with the broadcast and identify themselves with the community in terms of their ability to get recognized and to engage in community-specific ritual^33^.

### Monetizing Twitch content

It is through the interactive design described above that streamers profit from their broadcasts. If Twitch content is free, streamers also rely to a rather small extent on direct advertising, often using the absence of ads as an argument to promote their own streams^34^. Although there are methods outside the platform, such as sponsorship and other forms of advertising^35^, Twitch provides an internal monetization system highly linked to the social affordances the platform aims at developing.

As Cocq^22^ describes it, the work of the streamers is first and foremost aimed at their communities (“développement du capital communautaire” [community capital development]). Although limited ads revenue is possible on Twitch (therefore establishing some linear relation between viewership and payments), substantial monetization requires streamers to engage with their community, develop parasocial relationships through various means to build up their fanbase, and foster a sense of closeness to their viewers to push them toward paid features that generate the most part of the revenue (direct donation, subscription or “bits”).

Therefore, it is via the skilful exploitation of the social and relational affordances allowed by the platform that streamers engage in a peculiar form of two-sided parasocial interaction^36,37^, and are able to grow and consolidate a community around them.

This dimension of Twitch is the one most characterized in the literature. Hamilton^32^ characterizes Twitch streams as third places where viewers can interact and identify themselves around the streamer who acts as a central figure in the community. Other authors such as Sjöblom and Hamari^4^, Hu and colleagues^38^, and Gros^39^ argue that, from the viewers’ perspective, such sense of belonging to a community and interacting with members constitutes not only one of the main motivations to be on Twitch, but also for spending money on a stream. In this sense, streamers, consciously or not, create a sense of intimacy with their audience, and strive to flesh out their own community as an essential resource for extracting monetary gains from their broadcasts.

Woodcock and Johnson^23^ emphasize the centrality of the streamer’s affective work, ergo the necessity to engage emotionally, which means that streamers must constantly expose themselves and connect to their audiences in ways that are pleasing and that are felt as authentic, in order to elicit identification and trust. Similarly, Cocq^22^ emphasizes the need for the streamers to direct appreciation, gratitude, and attention to each individual viewer, and to keep personal information about regular viewers in mind so that their engagement is strengthened through the perception of a direct personal relationship. Jodèn and Strandell^16^ moreover show how, by means of the interaction ritual framework, streamers promote rapport specifically designed to enhance their credibility and reputation, their status within the community, and to secure viewers’ participation as a precondition for monetization, facilitated by multiple tools made available by Twitch, such as: channel points used to engage in gambling mechanics; specific badges visible in chat for paying subscribers, and so on.

Although these studies use different frames of reference and describe different mechanisms, they all aim at showing that the goal of streamers is first and foremost to engage viewers in the long term^40^ and to weaponize relational closeness and community making to drive their audiences into making payments, such as a subscription or direct donation, all of which are not mandatory, but rather entirely voluntary, and must therefore be supported by specific motivational cues.

This literature is therefore relevant in two different directions. On the one hand we can see how the affordances of Twitch should force streamers and audiences into a close relationship, putting a brake on the creation of one-to-many massive communication as typical of other social media. On the other hand, it is plausible that internal monetization favors small and medium-sized streamers rather than top-influencers as enjoyment is largely derived from the feeling of belonging to the community and having agency within the stream^13,41^.

Wolff and Shen^13^ already confirmed, to some extent, those claims. Their study however suffers from the limitations of a small sample in terms of streamers, games, and time-frame. Moreover, it does not take into account all the possible revenue sources from Twitch affordances as it relies upon data from the chat, and does not consider ads revenue as well as other different possible revenues that were being split between Twitch and the streamer.

### Growth and monetization

As mentioned above, Twitch should favor smaller scale, more direct interaction rather than massive streams that rely only upon broadcasting live action. This is due to the fact that the platform affordances point towards direct interactions, thus placing major emphasis on the sense of being a relevant member of a community and on the ability to influence the stream^42,43^.

In this sense, Twitch could appear as an “egalitarian” platform as compared to others, in that its affordances seem to favor the emergence of a multiplicity of smaller communities of highly engaged participants rather than massive channels, which, in a context like that of Twitch, would have a harder time in monetizing their audiences.

Considering that Twitch streamers rely on long term affiliation to build up their community and monetize it, we should observe relatively contained levels of inequalities across streamers. This idea is reinforced by the fact that both streamers and viewers have reasons to favor smaller audience size, and that it is precisely in the case of small community size that Twitch monetization affordances can be fully deployed.

If this reflection is not new^3,32^, it however becomes more relevant in the light of the recent pandemic-related growth of Twitch, that was led by a small number of massive channels.

The very existence of such massive channels might in fact be a signal that, despite the affordances as assessed at the micro-scale, the large-scale operation of Twitch could rather favor a restricted number of top streamers. Despite the presence of a multitude of active streamers, it is the very overabundance of channels and the lack of an algorithmic recommendation system that could jointly work in favor of those who already have gained visibility.

## Results

After the collection and aggregation of data, we kept 9523 streamers out of the initial pool of 10,000 top users by order of revenue. The omissions are mostly due to discrepancies and differences between the pseudonyms appearing in revenue data and the ones currently adopted by the streamers on Twitch).

As to the remaining streamers in our final dataset, we find earnings ranging from 9.5 million US dollars down to 23,520$ only, providing a sense of the order of magnitude of the disparities in revenues. After filtering the mismatches occurred during the scraping operation, we also found large inequalities in audience pools and retention.

### Inequalities and conversion

**Figure 1:**
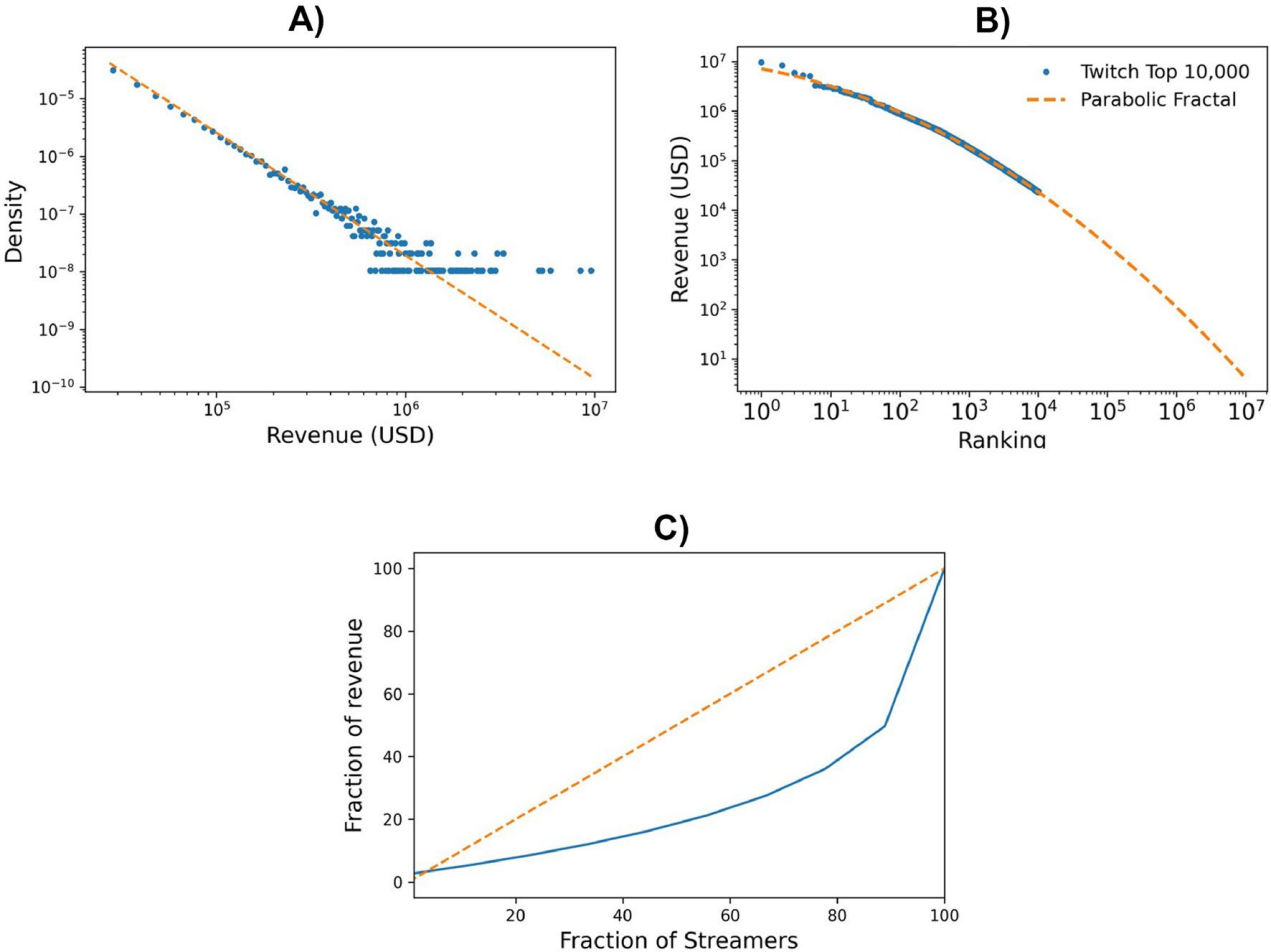
Inequalities in the streamers revenue from Twitch platform illustrated in three different ways. (**A**) The Revenue distribution for all 10,000 streamers between August 2019 and October 2021 (in US$). The distribution function appears long tailed and can be fit with a power law with exponent − 2.13. The Gini index associated with this distribution is $$\approx$$ 0.57. (**B**) Rank plot of Streamers revenue (in US$). Here, the curve is fit with a parabolic fractal function (orange dashed line, $$y=\log (x^{-b}) -c\log (x)^2 + norm$$, with parameters, *b* $$\approx$$ 0.259, *c* $$\approx$$ 0.0392, *norm* $$\approx$$ 15.78). Using the fit we could extend our estimate for the Gini index to all 9.2 million streamers present in the platform, for which we find the considerably larger value of $$\approx$$ 0.93. (**C**) Lorentz curve of streamers revenue, with perfectly equal distribution. Actual revenue distribution against perfectly equal distribution).

In order to measure such inequalities, we used the Gini index. The streamers were ranked according to the revenue received from Twitch over the 2019 2021 period. As it can be seen in Fig. 1B, the computed Gini index for the top 10,000 streamers revenue revealed significant inequalities (see Fig. 1, Gini value of 0.57). Moreover, projecting the analysis to the entirety of Twitch streamers using a parabolic fractal fit reveals a much larger level of inequality (Gini $\approx$ 0.93; Fig. 1B). This is a truly impressive value. If we consider more or less all the individuals having streamed at least once (Twitchtracker^27^), so that largely different situations are compared (from professional streaming to occasional amateurs streaming to their friends), clearly we expect that the level of inequality rises substantially. However, a level of inequality close to one remarkably illustrates the somewhat surprising scale of the difference in resource access of Twitch professional gamers as compared to amateurs.

**Figure 2:**
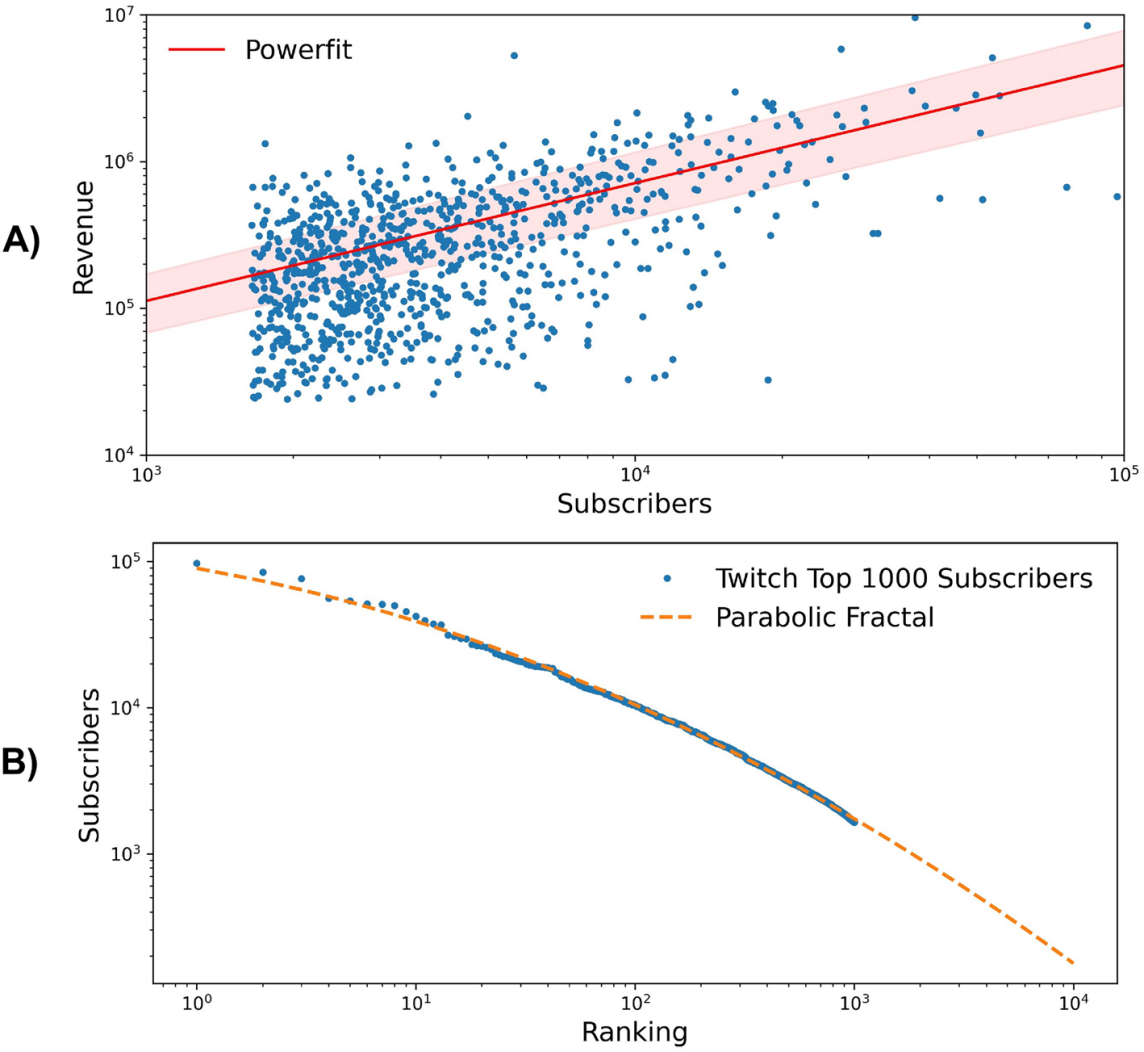
Relevance of paying subscribers in streamers monetization for the top 1000 streamers (by revenue). (**A**) Revenue in US$ for the top 1000 streamers, for which we could evaluate the paying subscribers count. The two quantities are naturally strongly correlated (Spearman $$\approx$$ 0.498; p-value $$\approx$$ 4.9 $$10^{-56}$$). The red solid line indicates a Power-law fit with exponent $$\approx$$ 0.8 ($$R^2$$ $$\approx$$ 0.38). The shaded area represents the 95% confidence interval. (**B**) Subscribers count against subscribers rank within the top 1000 streamers. The orange dashed line indicates parabolic fractal fit. Similarly as in Fig. 1, we can use the parabolic fractal to extend the estimate of the Gini index up to 10,000 streamers, for which we estimate a value of $$\approx$$ 0.6.

Likewise, looking at the disparities in the subscriber count for the top 1000 streamers and to its extension into a parabolic fractal for the top 10,000, we find similar levels of inequality (Gini: 0.63; Fig. 2B), highlighting the relevance of the size of subscriber pools for monetization, and corroborating the existing insights from studies of streamers’ monetization strategies.

As to the streamers’ ability to capitalize on their audience, we looked into the different conversion rates for streamers with different audience pool sizes. As suggested by Wolf and Shen^13^, in Fig. 2A we observe how top streamers convert their current subscribers into actual monetary gain relatively less, as highlighted by the sublinear scaling behaviour indicated by the power law fit with exponent $\approx 0.8$.

**Figure 3:**
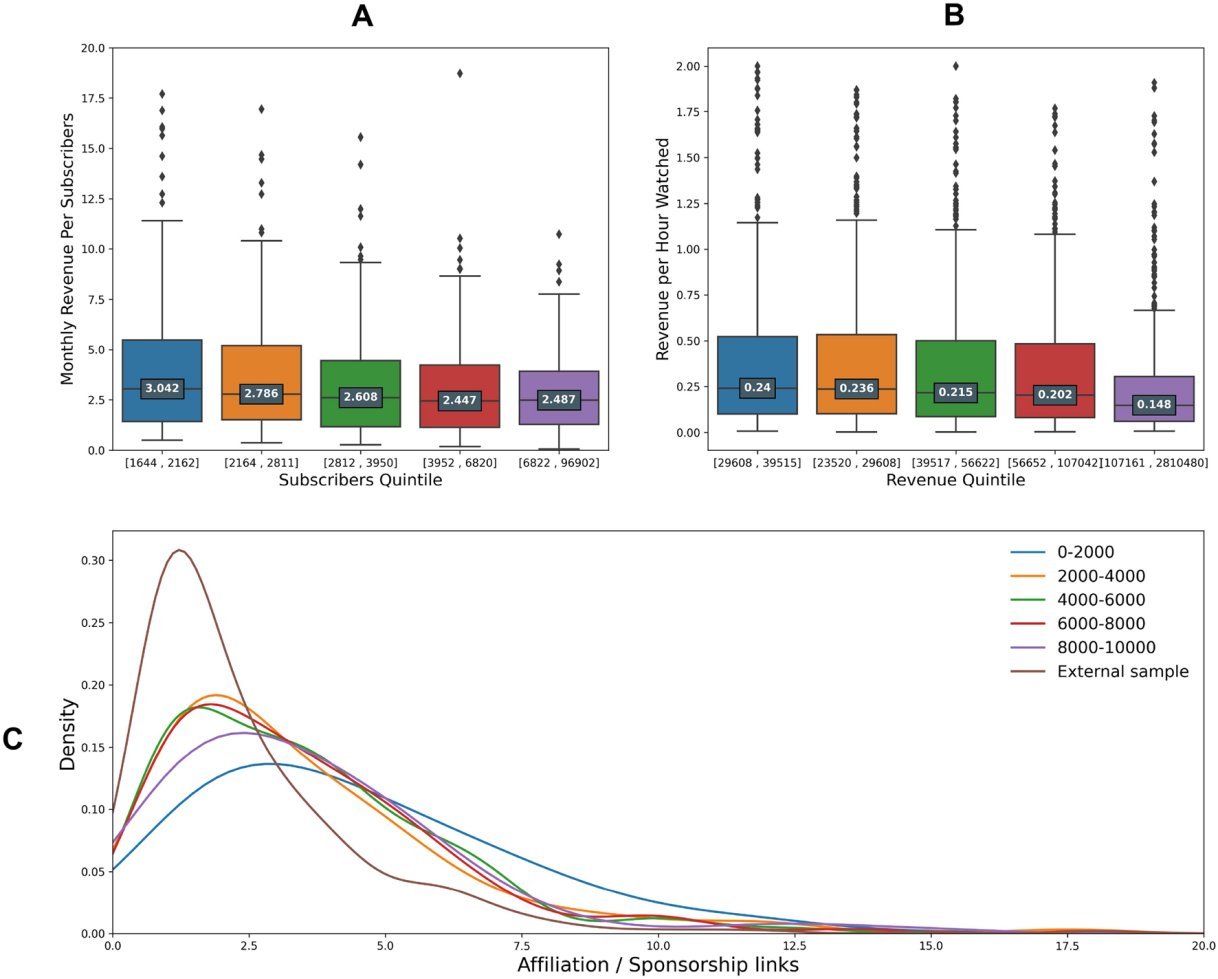
Audience rentability: Streamers grouped by revenue against type of revenue stream (subscriptions; ads; external monetization). (**A**) Box-plot of the streamers grouped in quintiles for number of subscribers against the average revenue per subscribers for a sample of 1000 streamers (source TwitchTracker^27^), illustrating the diminishing returns per sub of income in the Twitch platform. (**B**) Boxplot illustrating the revenue of the top 10,000 streamers grouped in quintiles against revenue per hours watched (hours collectively consumed by the entirety of viewers; source for hours watched: Twitch^45^). Users belonging to the richer group monetize less per hour watched. (**C**) KDE plot describing the number of affiliation or sponsorship URLs found in channel description (Directly bellow every stream page ; Source: Twitch^45^) for a sample of 1333 streamers grouped by revenue (external sample refers to streamers outside of the top 10,000). We see that the top 2000 streamers have an higher average (0–2000 $\approx$ 3.7; 2000–4000 $\approx$ 2.8; 4000–6000 $\approx$ 2.4; 6000–8000 $\approx$ 2.5; 8000–10,000 $\approx$ 2.7; External sample $\approx$ 2.2 ) as well as fatter tail distribution which is confirmed by looking at the standard deviation (0–2000 $\approx$ 4.1; 2000–4000 $\approx$ 2.8; 4000–6000 $\approx$2.4; 6000–8000 $\approx$ 2.6; 8000–10,000 $\approx$ 3.8; External sample $\approx$ 2.2).

Top streamers also capitalized relatively less, over the period, on their current subscribers (Fig. 3A). Furthermore it is easy to check that streamers with smaller revenues are relatively more able to convert non paying followers into paying subscribers (0.017 subscribers per follower for the lowest quintile against 0.008 for highest; see Supplementary Fig. 1). In a similar fashion, the more subscribers a streamer has as of February 2022, the less each individual subscriber brought over the course of the 2 years (Fig. 3A), even though streamers with higher subscribers count can strike better deals with Twitch regarding the distribution of the subscription money^44^.

Furthermore we can see from Fig. 3B that the higher the revenue, the less each hour watched by viewers translates into monetary gain, confirming the Wolff and Shen study^13^ which already showed (over a way smaller time-frame and streamer sample) that an increase in audience size led to diminishing monetary return from each viewer.

Using the URLs present in the channels description (directly accessible from the streams page), we compared the density of different types of redirection for a sample of streamers (grouped by revenue, as well as for a group of streamers external to our list, with revenues below those of the top 10,000) and showed that top streamers include significantly more links redirecting to affiliated brand and sponsors websites, even though we do not find significant differences for the other types of link (4 on average for the top 2000 and 3 for the others; one-way ANOVA; F-test $\approx$ 44.2, p-value $\approx$ 4.25 $10^{-11}$; see Fig. 3C). Moreover, the random sample (330) shows that streamers outside our sample have significantly less sponsorship links compared to the ones in our sample (on average only 2).

These results indicate that, if top streamers concentrate a large chunk of the revenue, from a “community capital” standpoint, they rank lower than the smaller streamers in our data-set. Consequently, top streamers succeed in monetization by making up for this deficit.

### Platform trends and viewership

In this section, we analyze the streamers’ ability to retain and grow their audience, as well as to follow and/or initiate platform trends as a means to get more insight into those inequalities which appeared at odds with the platform affordances.

**Figure 4:**
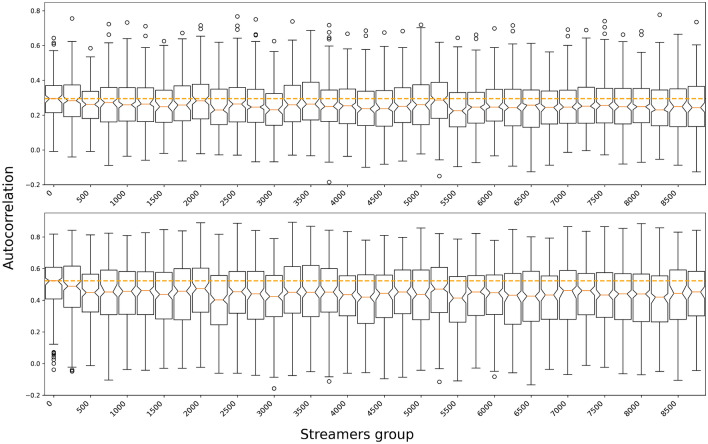
Autocorrelation coefficient for different streamers group. Box plot of the autocorrelation coefficient for ratio of time streamed (in minutes) against time streamed (in hours), i.e. time collectively consumed for each minute streamed, for different streamers group (aggregated by revenue; top 0–250; 250–500 etc...) and for different time lags (weekly, biweekly). The orange dashed line represents the median coefficient for the first group. The plots are showing that the the streamers in the top 250 consistently present the highest autocorrelation. In other words, the ratio between broadcast time and visibility displays a more stable trend for top streamers.

First of all, we analyze the auto-correlation in the hours watched per minute (i.e., the ratio between watching time and streaming time) returns for streamers ranked by Twitch revenue for different order (i.e the predictive power of t1 over t2, t3 etc...). In Fig. 4 we illustrate the values for autocorrelation for shifts of 2 and 4 weeks, therefore showcasing how the previous week evolution explains the next week evolution. We can see that top streamers display, on average, a higher level of correlation or, in other words, a more stable trend. Moreover, averaging the results for the different groups, we find that the top streamers consistently feature the highest level of auto-correlation (up to 4 week lags, Supplementary Fig. 2A, that is, a more consistent trend. Finally, the higher the lag the more consistent the trend, most likely due to the general growth of the platform leading to consistently good returns by streamers revenue group), but we can also observe that top streamers maintain the highest level of auto-correlation and are the most stable (see Supplementary Fig. 2B).

**Figure 5:**
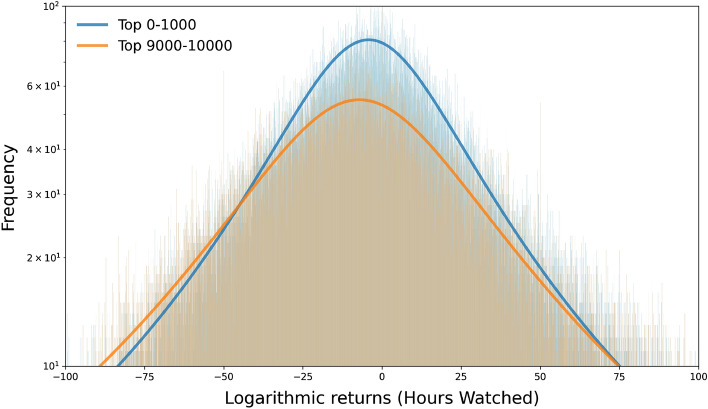
Histogram of the hours watched logarithmic returns (weekly). Histogram of the Logarithmic returns of the hours watched and the fitted Cauchy curve for the Top 0–1000 (Location $\approx$ 3.15, Scale $\approx$ 29.4) and Top 9000–10,000 streamers (Location $\approx$ − 7.88, Scale $\approx$ 39.61). These fits show that top streamers manage to keep a more stable viewership through time whereas the low end of the distribution has a more volatile visibility.

We then find a consistent result using methods borrowed from the study of financial time series^46^. We compare the variation in the hours watched, which appeared to be a great metric in order to estimate streamers visibility on the platform. By grouping the returns per hours watched for the fitting Cauchy curve we show that top streamers experience less variability (we observe the narrowest curve Scale $\approx$ 29.42). Moreover, the top 1000 streamers group is the closest to a stationary trend, suggesting that if top streamers may face variability in their audience they nonetheless have a better ability to recover from a sudden loss of viewership and stabilize or even grow after a surge in visibility (see Fig. 5).

We therefore see, using two different methods, that top streamers, despite that their massive audience should be harder to retain, face less variability than smaller ones as to their capacity to keep their viewers engaged or to renew their commitment over time. Therefore, even if on a weekly or biweekly basis (lag 1) we do not find a clearly stable pattern, over time it results that top streamers better manage their audience retention or renewal. Consequently, this should allow them to maintain their position at the top.

Using the ranking dynamic model developed by^47^ we were able to show that top streamers not only maintain their audience but also maintain their position at the top. The model allows to sum up different characteristics of the evaluative ranking system into a single parameter. Looking at the fitted parameter^47^, we can confidently say that the system is extremely stable. Twitch’s rankings range from 0.13 to 0.20, which is comparable to the results found for word usage in different languages (French/Russian/German = 0.18) or to some of the natural phenomena studied by Iñiguez and colleagues, while showing a clear departure from the parameters range found in most “societal” datasets.

**Figure 6:**
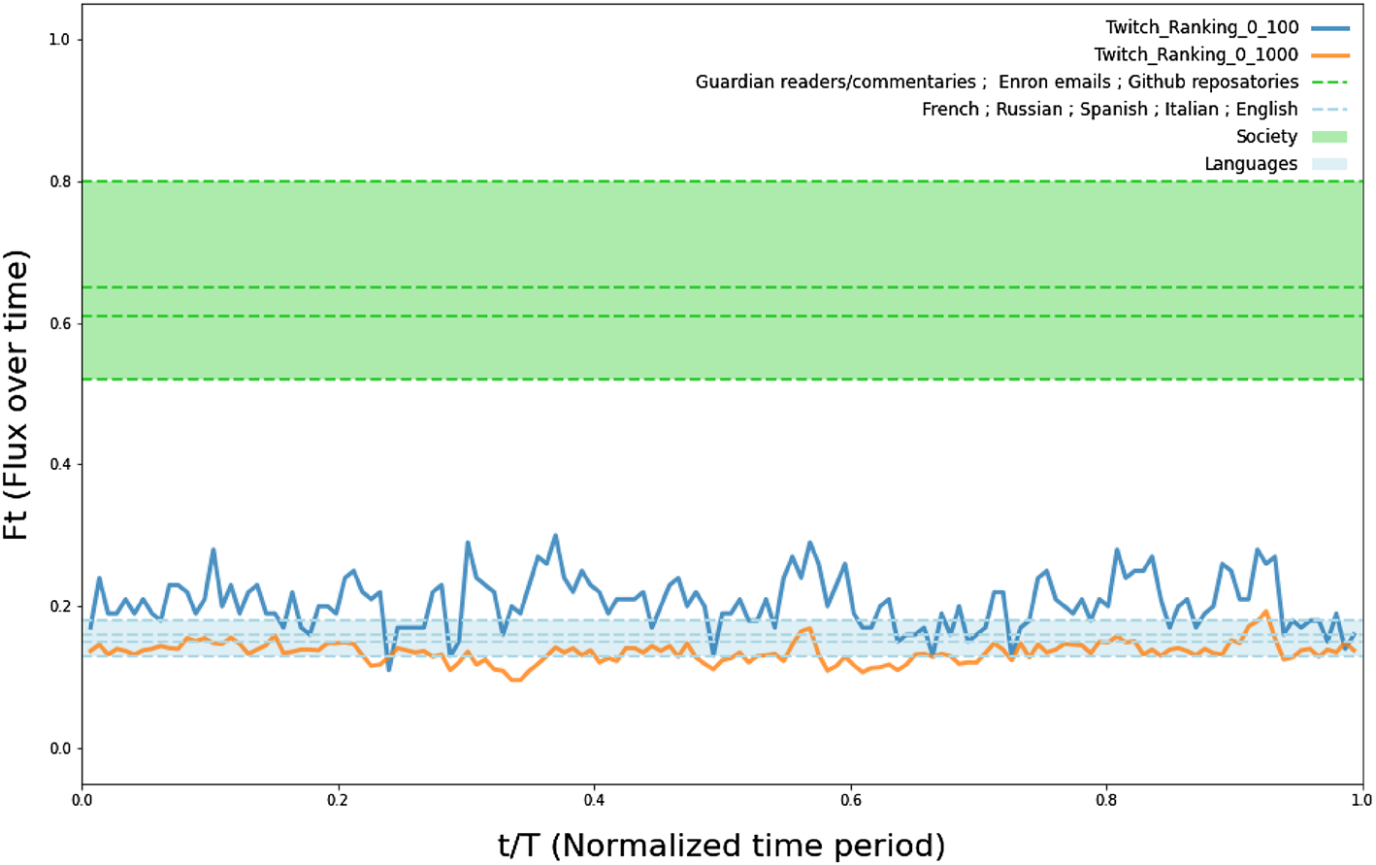
Time flux (Ft) plot over normalized time period (t/T) for Twitch (solid lines) and parameters from original study (bands, dashed lines): Flux defined as the probability that an element present in the list at T0 leaves at T1 over normalized time period: Twitch top 100: F $\approx$ 0.2; Twitch top 1000: F $\approx$ 0.135; Society datasets (Guardian readers: F $\approx$ 0.80; Github repositories: F $\approx$ 0.65; Guardian commentaries: F $\approx$ 0.61; Enron emails: F $\approx$ 0.52); Language (word usage) datasets (French: F $\approx$ 0.18; Russian: F $\approx$ 0.18; Spanish: F $\approx$ 0.16; Italian: F $\approx$ 0.15; English: F $\approx$ 0.13). This shows that the Twitch ranking dynamics resemble that of more relatively stable datasets such as language related ones rather than more variable, social systems related ones.

Specifically, the fitted models show that the probability of streamers in the list to drop below the threshold value for staying in the list at any round is about 0.2 for the top 100 and 0.13 for the top 1000 (Fig. 6). Moreover, in the supplementary plot, we can see that 50% of streamers in the list appear from the very early moments, and the higher the position in the list, the lower the probability of dropping out (see supplementary Fig. 4). Therefore, the model reveals higher volatility, for every given cut, in the low end of the distribution, showing that streamers in top positions have a lower probability to leave the list at time t + 1 (weekly increments), whereas the lower the position in the list, the higher the probability to drop out after n weeks. A top streamer status, in addition, is very stable. In particular, top streamers are likely to appear in the ranking since the very beginning (this is the case for more than 50% of them).

Our results show that not only the top streamer status is highly stable but that Twitch, as a system, is highly stable as well. Therefore we can conclude that since top streamers maintain their audience and also stay at the top, they must be able to better exploit platform features that allow them to maintain such a position.

Moreover, one should note that the discrepancies between the parameters for the top 100 and top 1000 appears to be the result of size effects (see Supplementary Fig. 3, which displays a much more volatile and higher average flux), showing that even if the top is highly stable, the larger the pool, the more challenging for streamers to maintain their position in the ranking.

**Figure 7:**
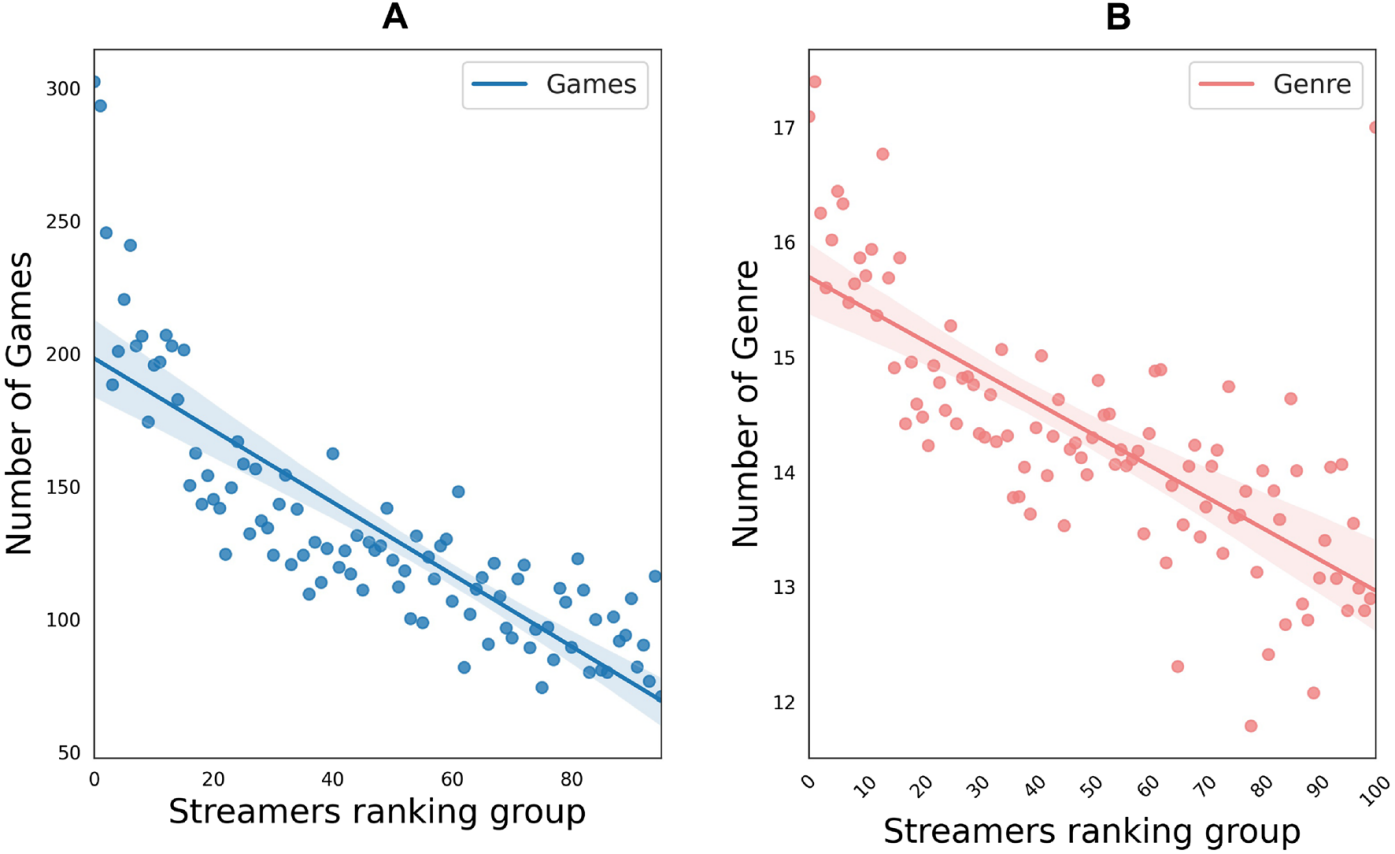
Average number of games/genre played against streamers revenue rank. (**A**) Average number of games played for at least 1 h—evaluated as the category where the streamer placed his stream (which in general maps to a single game)—for each streamers group (100 groups; aggregated by revenue) from 2019 to 2021 (source: IGDB^48^). Spearman Correlation $\approx$ 0.89|P $\approx$ 3.26 $10^{-34}$; R$^2$ $\approx$ 0.48. (**B**) Average number of game genres played (source: IGDB^48^) for each streamers group (100 groups; aggregated by revenue). Spearman Correlation $\approx$ 0.76|P $\approx$ 9.05 $10^{-9}$; R$^2$ $\approx$ 0.47. The plots show the relationship between viewership and content diversity: top streamer appears to have more diversified, likely trend following, behavior.

Finally, as mentioned above, we also collected the gaming activity by the different streamers. Analyzing these data, we were able to show that top streamers have more diverse activities in the platform, either in terms of games played across the period (2019–2021; Spearman $\approx$ 0.89; p-values $\approx$ 1.5 $10^{-36}$; Fig. 7A) and of different genres of games^48^. We get extremely similar results in both cases (Spearman $\approx$ 0.76; p-values $\approx$ 9.05 $10^{9}$).

Thus, for a given streaming time, top streamers play significantly more games (or a broader range of activities on stream) (Spearman $\approx$ 0.89) belonging to a wider variety of genres^48^ (Spearman $\approx$ 0.76), confirming the idea that top streamers are offering a more diverse content than others (see Fig. 7) therefore giving them the opportunity to reach a wider audience, renew their viewership, ride the hype on the platform and consequently achieving more direct monetization through ads and opportunities to nudge more people toward paying features.

## Discussion

The purpose of our paper was to characterize revenue inequalities in the Twitch platform. Based on the information provided by the literature, we hypothesized that, due to its affordances that favor long-term affiliation and high level of engagement, Twitch would display relatively small levels of inequality.

Contrary to our initial expectation, we instead observed that the platform reproduces the typical structure of digital platforms, with power law-like distributions of both revenue and viewership. Even if we found some factors with a potential countervailing effect on inequalities, namely that streamers with bigger audiences face relatively less opportunities to convert their activity volume into revenue per activity unit through the platform, we also showed that top streamers generated more revenue stream while monetizing their content.

To explain these results, we proposed the hypothesis that top streamers had a better ability to retain their audience thanks to a better capacity to adapt to changes. This conjecture appears to be supported by the fact that top streamers face less variability, maintain their audience through the period of observation, have a fairly stable ranking dynamic and play more games from more different genres than others.

Our results thus question the relevance of an ’algorithm-free’ approach such as that of Twitch when it comes to inequality reduction. The real ability provided by Twitch for new streamers to make use of the platform affordances to derive monetary gain from their activity is more limited than one could expect. In the absence of an algorithmic discriminant capable of sharply altering user behavior, the mismatch measured by our analyses is explained by referring to the old concept of information overload. As pointed out by Simon in the pre-digital era^49^, with an excessive supply of information what becomes scarce are time and cognitive resources: this inevitably leads people to choose on the basis of some heuristics that allow an optimization of aims (acquiring the information being sought) and means (saving time). Among these heuristics, one of the most used when facing a dilemma of over-abundance is that of selective trust: the information already socially validated (in our case, opting for Twitch top streamers) reduces the risks of a cognitively expensive and lengthy exploration. This paves the way to a rich-get-richer effect within a relational sphere of influence (namely, that of streamers and subscribers), that can radically alter the expected distribution of resources under less resource-constrained informational conditions.

Regarding our methods, we made the choice to substantially enrich the initial dataset via scraping and other forms of data collection. We made use of classical tools of computational social science, economics and complex systems methods to analyze the inequalities across streamers, and proposed an explanation as to why our result did not reflect what was expected from the literature.

We nonetheless must observe that, if the data leaks provided a unique opportunity to look behind the curtain and translate inequalities in viewership into revenue ones, such leaked data present some limitation. First and foremost, there are ethical and legal limitations associated to the nature of the data^50^, that in our case have been avoided by accessing top 10,000 revenue ranking data derived from the leaked data^31^ which have been included in publicly available sources such as several websites^31^ and web articles^51,52^, and focusing our analysis only on the revenue values and the associated position in the ranking. The streamers pseudonyms were only used to link these values to other measures, a passage that represented one of our main problems with those data because pseudonyms may change across time and thus across different datasets, which forced us to adjust data and at times to discard them altogether. Moreover, we must acknowledge that we only had partial information about the streamers’ revenues, which forced us to extrapolate and complete the data using imperfect methods. And finally, as for most digital sources, the information we have, even if rich, is still partial and some finer analysis regarding audience structure and characteristics, or streamers’ activity, could be beneficial.

Finally, despite their limitations, our results raise a more general question regarding inequality reduction on online platforms. As mentioned, Twitch aims at developing highly engaged communities, and therefore made a conscious choice not to leverage upon algorithmic recommendation systems. Such choice, however, leads to low discovery affordances and allows top streamers to capture much of the audience, trend after trend. If authors like Spilker et al.^53^ note that viewers can actively seek smaller streams with higher social connectivity, it appears that they face a known situation of information overload or, as Schwartz^54^ put it, a paradox of choice, where in a situation of abundance of options, the consumer can be paralyzed in front of it and even face detrimental consequences when trying to maximize their choice satisfaction. We therefore can only assume that many, dealing with such platform design, don’t try to optimize but rather settle for a “good enough choice” by going for top streamers rather than indefinitely scroll for the perfect broadcast, which would imply giving up considerable entertainment time for less enjoyable search activity. How to balance, then, the inequalities caused by algorithms and those embedded in human choice behavior? Despite the egalitarian rhetoric that has been built around the platform, Twitch does not seem to provide a viable solution to this pressing issue.

## Methods

### Data sources

In this paper, we used Twitch leaked data regarding the earnings received by streamers from Twitch over the 2019 to 2021 time period^31^. We have to remark that even if the earnings mentioned in the leaked document were not directly confirmed by the Twitch owner company Amazon, their reliability was however corroborated by multiple streamers. However, we further enriched our study with other data coming from different sources (namely Twitch Tracker^27^ and Stream Charts^55^). In order to complement our result with data relating to audience sizes and better characterize the dynamics of community formation, we collected time series data using stream charts as well as data relating to the current audience and the streamers activity^27,45,56^. This triangulation of sources aims at investigating the actual levels of inequalities in revenues, audiences and the socio-structural processes underlying such inequalities.

To quantify such inequalities and analyze the observed dynamics leading to the current situation, we used a classical inequality metric such as the Gini index, as well as tools used in the study of financial time series that can unveil the differential evolution of the sizes of streamers viewership. Finally, to further characterize the viewership dynamics over the two years period of observation, we employed the ‘Ranking dynamics’ method proposed by Iñiguez and colleagues^47^.

### Data collection

The first set of data collected for our study derives from a leak of Twitch data happened in 2021. Even if those leaks contained multiple types of data, in this paper we only consider the information relating to the streamers’ revenue from August 2019 to October 2021 that has been computed by third parties and reported in several websites^31^ and publicly discussed in the press^51^. It is important to stress that the leaked data only consider the money flows received from Amazon, the Twitch parent company, and thus only represent a part of the streamers’ actual revenues.

To complement these data and further explore the underlying community formation processes and growth dynamics we also collected the weekly evolution of different audience metrics as well as the game activity for every streamer featured in our data, and the associated audience time series for a sample of such games.

Through these data we are able to characterize audience features in terms of followers (individuals having clicked the follow button), hours watched (the number of hours collectively watched by viewers), average viewers (average number of concurrent spectators over a set period of time), active time (the amount of time the stream is ‘on’ for a set channel) and streamers activity (the different games played through the course of each year). If audience data correlate both indirectly (more viewers and visibility offer more opportunity for monetization) and directly with revenue (through ads) they have been complemented by sources relating directly to the revenue through the leaks (revenue distributed by Twitch for top 10,000 streamers), subscriber count (viewers paying a monthly subscription) for a sample of streamers and the specific URL (classified into different categories via a rudimentary method based on keywords) present in the channel description (text, images and URLs embedded in the stream page) for a sample inside and outside our list (the latter selected randomly from streamers active during May 2022).

Data collection was conducted between January and April 2022. In Table 1 we list the different sources and their coverage, which is in general limited to the period between 2019 and 2022.

**Table 1:** List of data sources.

Data				
Information	Source	Collection period	Variable	Covered period
Streamers revenue	Twitch leaks^31^		Revenue (US$)	August 2019–October 2021
Streamers engagement	Twitch API^45^	February 2022	Status; followers; views	February 2022
Streamers subscribers (top 1000)	Twitchtracker^27^	February 2022	Subscribers count	February 2022
Streamer audience	StreamCharts^55^	March–April 2022	Hours watched; average viewers; airtime	June 2019–March 2022
Streamers games	Twitchstats^56^	April 2022	Games	2019–2022
Games audience (top 500)	Twitchstats^56^	April–May 2022	WatchTime	June 2019–March 2022

All data sources, with the exception of the Twitch leaks, come, indirectly, from the Twitch API. The other websites mentioned only function as data aggregator (since the Twitch API only returns live information) allowing to create time series for the different metrics. Whereas every aggregator has a different way to collect data, they nonetheless typically make a collection every 15 min and then aggregate the different metrics on an hourly or daily basis.

### Measurement

In order to determine inequality levels of the variables of interest in the platform, we calculated the Gini coefficient for three fixed metrics (revenue, followers, subscribers) and then we fitted a power law and a parabolic fractal curve to extend the top streamer list. The Gini coefficient ranges from 0 to 1 and describes the distribution of a variable in a population where 1 denotes a situation of perfect inequality (a single individual concentrates all the resource) and 0 a situation of perfect equality (every individual has the same level of the resource).

Moreover, in order to characterize the dynamics of audience pools we make use of classical methods used in the study of financial time series, such as those in^46^ and^57^. These methods, that classically allow to measure the volatility in stock markets, allowed us to assess the growth of the audience pools of different streamers.

To confirm the stability in the top tier of streamers and in Twitch as a whole, we used the ranking dynamic model proposed by Iñiguez and colleagues^47^ in order to showcase that in addition to stabilizing their audience, top streamers also maintain their elite status.

Finally we compared the diversity in activity as a function of the ranking, using the number of games and variety of genres played through the period.

## Supplementary Information

Supplementary Figures.

## Data Availability

Data regarding games played and publicly available statistics regarding streamers^27,45,56^ are available upon request from the corresponding author. Restrictions apply to the availability of these data. Data regarding streamer revenue^31^ and viewership time series^55^ are obtainable by an interested reader directly from the source.
