# Supplementary material for: Monetization in online streaming platforms: an exploration of inequalities in Twitch.tv
Source: Sci Rep. 2023 Jan 20;13:1103. doi: 10.1038/s41598-022-26727-5 (PMC9852796; doi:10.1038/s41598-022-26727-5)
Supplement: Supplementary file 1 — Supplementary Figures. [file 41598_2022_26727_MOESM1_ESM.pdf]

Supplementary Information for:
Monetization in online streaming platforms: an exploration of inequalities in twitch.tv

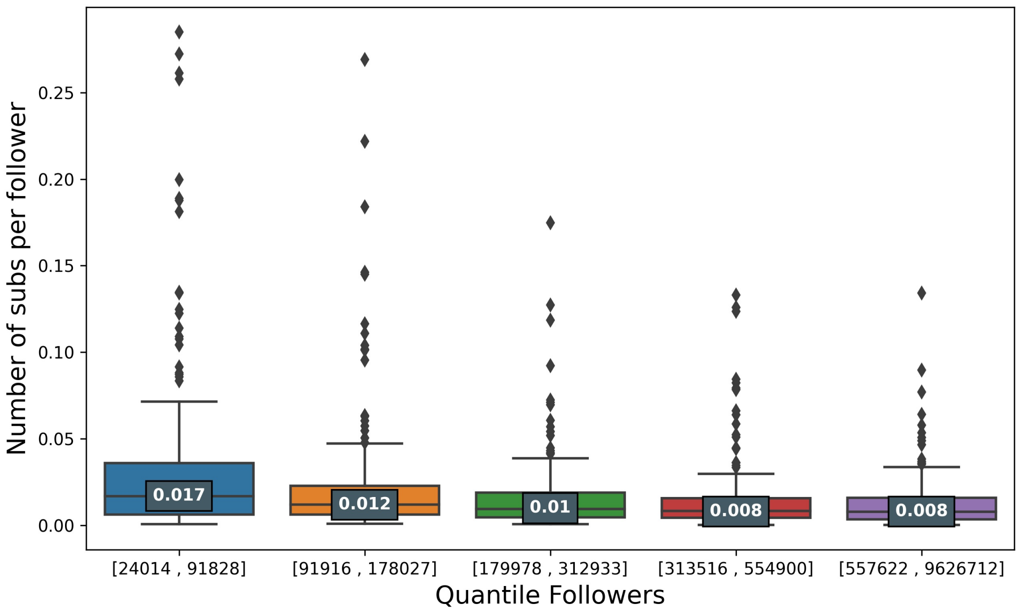

Supplementary Figure 1: Box-plot of the streamers grouped in quintiles for revenue against the fraction of follower subscribing to the channel (Source TwitchTracker [27]). The figure illustrates the conversion (from follower to paying subscribers) difficulties of top streamers.

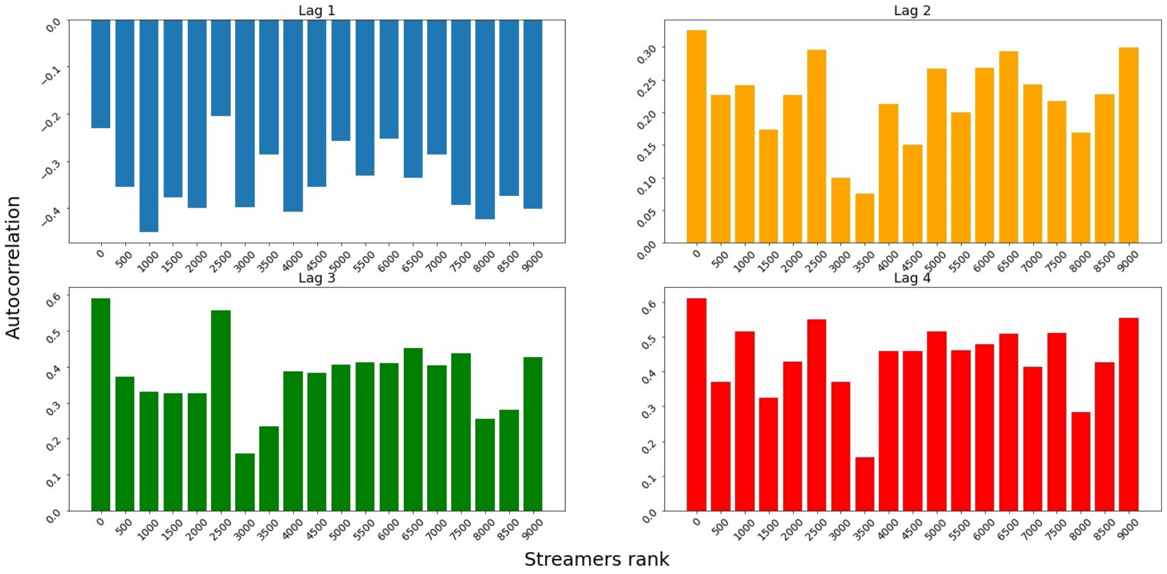

Supplementary Figure 2A: Auto-correlation coefficient for different streamers grouped by hours watched per minutes streamed returns (averaged). The plot shows that top streamers consistently have higher coefficient than the other groups.

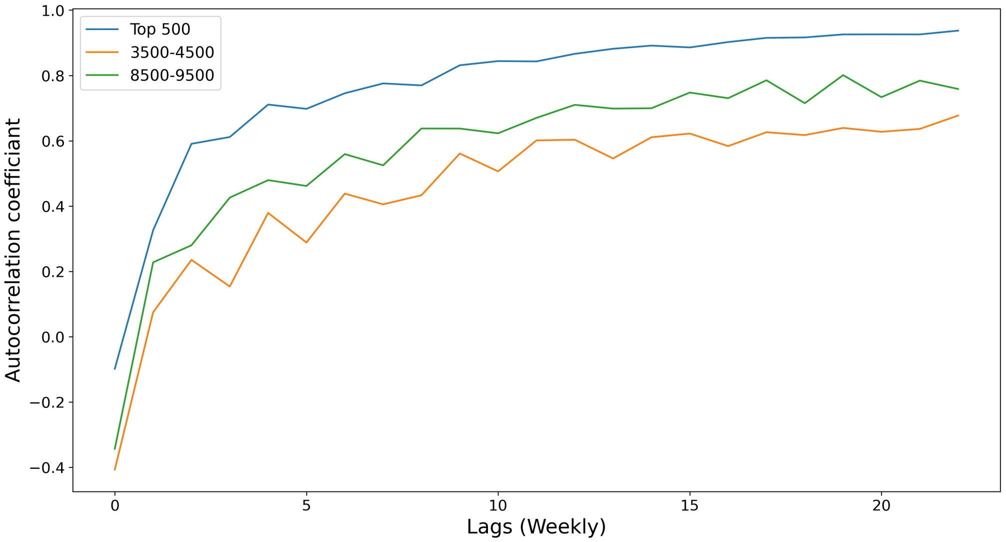

Supplementary Figure 2B: Auto-correlation coefficient for different streamers grouped by hours watched per minutes streamed returns (averaged). We see that, most likely due to the general growth of the platform, the auto-correlation levels are high and remain stable for all groups but we also see that the most stable one (i.e the group benefiting the most and most consistently from this growth) is the top 500 streamers.

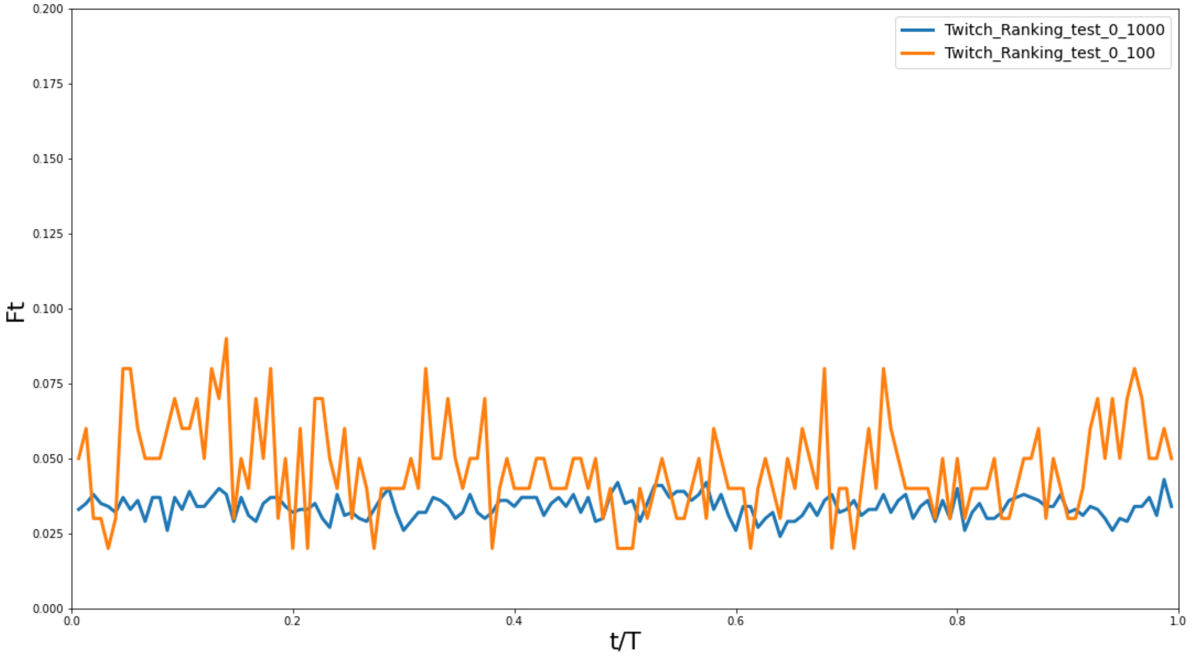

Supplementary Figure 3: Test of the effect size on the model. The data has been generated using similar differences in the orders of magnitude for T0 and the variation through time is random (from a normal distribution centered around the previous value). We see that expanding the list results in a more volatile flux and an higher model parameter (100 elements $\approx$ 0.03; 1000 elements 0.05.)

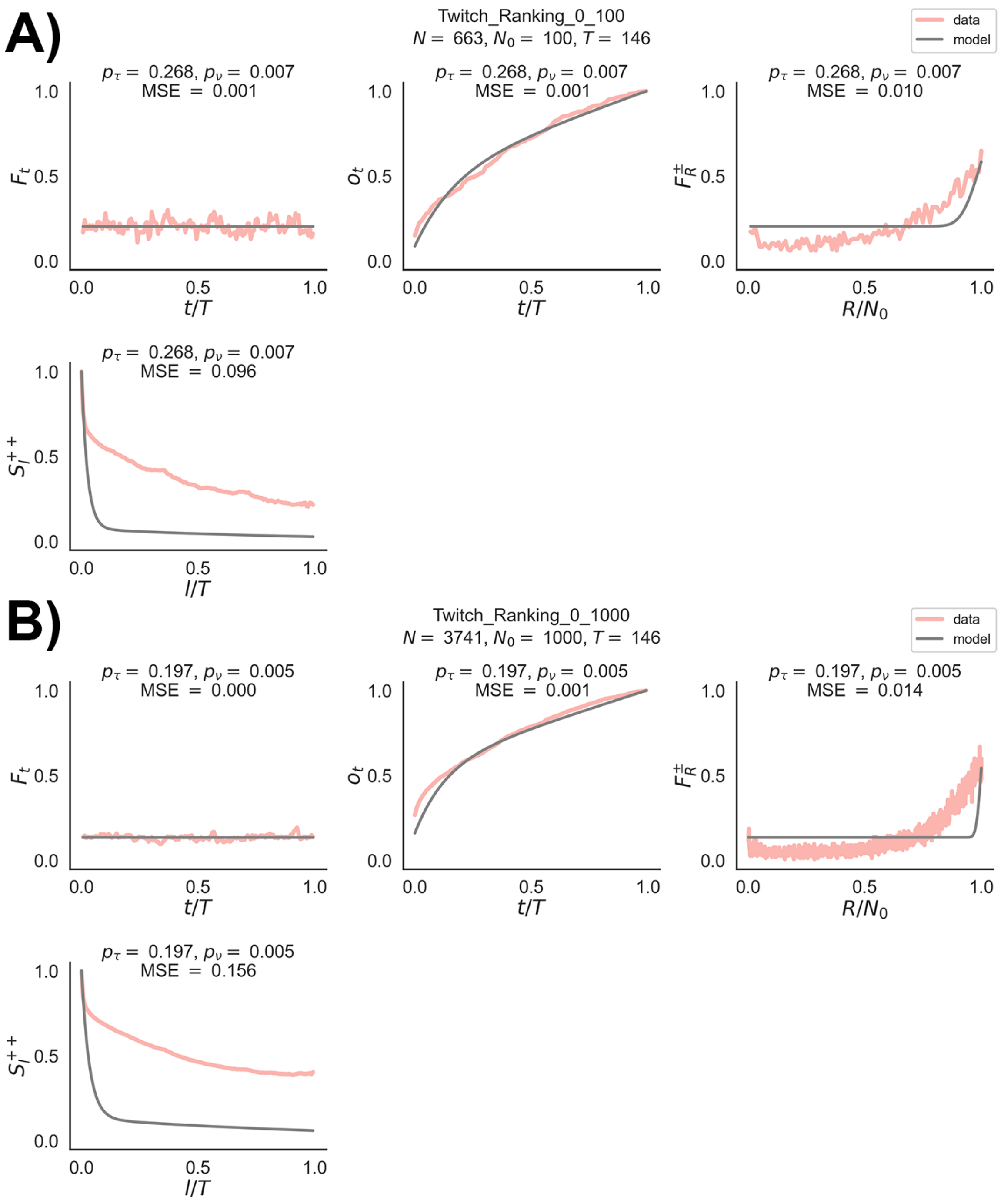

Supplementary Figure 4: Ranking dynamic fits for top 100 and top 1000. From top left to bottom the plot represents the flux (probability that an element leaves the ranking at time t, turnover (fraction of elements which appeared at time t), out-flux (probability that an element at rank (R/N) will leave the ranking at time t+1) , inertia (probability that an element in the top of the ranking will stay in the top after t+n observations).
